# Human malaria diagnosis using a single-step direct-PCR based on the *Plasmodium* cytochrome oxidase III gene

**DOI:** 10.1186/s12936-016-1185-x

**Published:** 2016-02-29

**Authors:** Diego F. Echeverry, Nicholas A. Deason, Jenna Davidson, Victoria Makuru, Honglin Xiao, Julie Niedbalski, Marcia Kern, Tanya L. Russell, Thomas R. Burkot, Frank H. Collins, Neil F. Lobo

**Affiliations:** Eck Institute for Global Health, University of Notre Dame, Notre Dame, IN 46556 USA; Australian Institute of Tropical Health and Medicine, James Cook University, Cairns, QLD 4870 Australia

**Keywords:** Malaria, Blood spots, 18s-rRNA, Cytochrome oxidase III, Diagnostic, PCR, Direct PCR, *Plasmodium*, *Plasmodium ovale*, Submicroscopic infections

## Abstract

**Background:**

Nested PCRs based on the *Plasmodium* 18s-rRNA gene have been extensively used for human malaria diagnosis. However, they are not practical when large quantities of samples need to be processed, further there have been challenges in the performance and when interpreting results, especially when submicroscopic infections are analysed. Here the use of “direct PCR” was investigated with the aim of improving diagnosis in the malaria elimination era.

**Methods:**

The performance of the *Plasmodium* cytochrome oxidase III gene (COX-III) based novel malaria detection strategies (direct nested PCR and direct single PCR) were compared using a 18s-rRNA direct nested PCR as a reference tool. Evaluations were based on sensitivity, specificity and the ability to detect mixed infections using control blood spot samples and field collected blood samples with final species diagnosis confirmation by sequencing.

**Results:**

The COX-III direct PCR (limit of detection: 0.6–2 parasites/μL) was more sensitive than the 18s-rRNA direct nested PCR (limit of detection: 2–10 parasites/μL). The COX-III direct PCR identified all 21 positive controls (no mixed infections detected) while the 18s-rRNA direct nested PCR identified 18/21 (including four mixed infections). Different concentrations of simulated mixed infections (*Plasmodium vivax* and *Plasmodium falciparum*) suggest that the COX-III direct PCR detects only the predominant species. When the 18s-rRNA direct nested PCR was used to detect *Plasmodium* in field collected bloods spots (n = 3833), there was discrepancy in the results from the genus PCR (16 % positive) and the species-specific PCR (5 % positive). Further, a large portion of a subset of these positive samples (93 % for genus and 60 % for *P. vivax*), did not align with *Plasmodium* sequences. In contrast, the COX-III direct PCR clearly identified (single bands confirmed with sequencing) 2 % positive *Plasmodium* samples including *P. vivax*, *P. falciparum*, *Plasmodium malariae* and *Plasmodium ovalewallikeri*.

**Conclusions:**

The COX-III single direct PCR is an alternative method for accurate detection of *Plasmodium* microscopic and submicroscopic infections in humans, especially when a large number of samples require screening. This PCR does not require DNA isolation, is sensitive, quick, produces confident/clear results, identifies all the *Plasmodium* species infecting humans, and is cost-effective.

## Background

Significant progress has been made in the global fight against malaria through the scaling-up of indoor residual spraying (IRS), insecticide-treated bed nets (ITNs), rapid diagnostic tests (RDTs) and artemisinin-based combination therapy (ACT) [1, 2]. As malaria endemic regions approach pre-elimination, a switch from passive detection to active screening of large numbers of samples will be needed to find and treat asymptomatic infections [3]. Design and implementation of sensitive diagnostic tools are thus needed to detect asymptomatic infections that would otherwise go unnoticed and contribute to the persistence of malaria transmission [4].

Estimates of human malaria prevalence and incidence are dependent on the sensitivity of the diagnostic method. Under field conditions, experienced microscopists typically detect parasitaemias of 50–100 parasites/µL [5], but misdiagnosis is common with mixed infections and low parasitaemias are often missed. In areas where microscopy is not feasible, rapid malaria diagnostic tests (RDTs) target the lactate dehydrogenase and aldolase proteins found in all *Plasmodium* species (Pan-specific malaria RDT). The histidine-rich protein 2 (HRP2) based RDT is specific for *Plasmodium falciparum* [6]. These RDTs have a limit of detection around 200 parasites/µL and are influenced by other factors such as deletion of target genes, the method of storage and handling of RDTs, concentration of proteins in blood, the manufacturer and subjectivity in interpretation of results which may affect the accuracy of the tests [7–11].

Molecular tools have also been utilized for malaria diagnosis and have improved the capacity to diagnose submicroscopic infections [12, 13]. These are defined as a low-density of *Plasmodium* parasites in blood that are unlikely to be detected by conventional microscopy. A meta-analyses of 38 studies comparing the sensitivity of microscopy and PCR for malaria diagnosis demonstrated that an average of 69 and 56 % of *Plasmodium vivax* and *P. falciparum* infections, respectively, identified as positives through PCR were not detected by microscopy [12].

Since the early 1990s, PCR-based diagnostic methods targeting the small subunit rRNA gene (18s-rRNA) have been routinely used for the detection of human *Plasmodium* species [14–16]. A nested PCR using this gene is considered the standard for molecular-based malaria diagnosis [3]. The reported diagnostic sensitivity of tests based on the 18s-rRNA target ranges from 1 to 10 parasites/µL [16–18]. This nested PCR, as well as the mitochondrial *cytochrome b* [19, 20], antigenic genes such as *stevor* and *msa*-*2* [21], PgMt19 and PfMT869 mitochondrial regions [22], and the *Pvr47* and *Pfr364* genes [23, 24], have allowed screening of field samples for epidemiological studies and characterization of submicroscopic malaria infection. However, the level of sensitivity is variable depending on the method used and characteristics of the target gene.

Recently, a novel technique called “direct PCR”, was developed based on DNA amplifications using a genetically modified DNA polymerase with rapid performance in presence of strong inhibitors from blood [25]. As a consequence, DNA extraction and PCR amplification can be combined in a single step, saving resources and time, while minimizing human error and contaminations. Direct PCRs has been used to diagnose infectious diseases [17, 26], to detect molecular markers of cancer [27], to identify human pharmacogenomics markers for HIV [28], for forensic genotyping [29] and was applied to malaria diagnosis using the 18s-rRNA following a nested PCR approach [17, 30].

Nested PCRs are still routinely used to identify *Plasmodium* genus and species. This may be impractical for diagnosis in the malaria elimination era, where the proportion of submicroscopic infections are expected to increase [4, 31] and larger number of samples will be required for human malaria epidemiology studies. The main goal of this study is to provide faster and more sensitive molecular tools to improve malaria diagnosis in the era of malaria elimination programmes. The use of a direct single PCR based on the *Plasmodium* cytochrome oxidase gene (COX-III) was investigated and compared against the 18s-rRNA direct nested PCR for malaria diagnosis.

## Methods

### Parasite reference strains and positive control blood samples

Malaria parasites (in blood spots or as extracted DNA) from *P. falciparum* (HB3 strain), *P. vivax* (AMRU1 and MIAMI strains), *Plasmodium ovale* (I.D 5463) and *Plasmodium knowlesi* (Malayan strain) were provided by Michael Ferdig (University of Notre Dame, USA), Robert D. Cooper (Australian Army Malaria Institute, Australia) and the Malaria Research and Reference Reagent Resource Center (MR4) USA, Harald Noedl (Medical University of Vienna, Austria), and John W. Barnwell (Centers for Disease Control and Prevention, USA), respectively. Microscopy was used to determine parasite densities of *P. vivax* (AMRU1) obtained from *Aotus* monkeys and *P. falciparum* (HB3) from continuous culture (about 70 % rings), which were serially diluted and blotted on filter paper ranging from 500 to one parasites/μL and 500–0.06 parasites/μL, respectively. Twenty-one malaria positive (as diagnosed by microscopy) blood samples were included as positive controls.

### Field human blood samples collection

A total of 3833 finger-prick human blood samples were collected in the Western Province of the Solomon Islands. Blood was spotted onto filter paper (Whatman 3 MM, Whatman International, Maidstone, England) and air dried at ambient temperature before storage in individual plastic bags with silica gel. Ethical approval for the study was obtained from University of Notre Dame, USA (FWA 00002462); National Health Research and Ethics Committee, Solomon Islands (HRC13/14 and HRC14/16); and the James Cook University Human Research Ethics Committee, Australia (H4915). Informed consent was sought from all the subjects who provided blood samples.

### Preparation of blood spots

For each sample, two 1.5 mm diameter samples of dried blood (equivalent to 3–5 µL of blood) [32] were placed in the well of a 96-well PCR microplate (Axygen Scientific, Union City, CA). Each plate included two positive controls (*P. vivax* or *P. falciparum*) and three negative controls (two blood spots from a known malaria negative donor and one no-template reaction control). To remove haemoglobin, 70 µL of molecular biology grade water (Cellgro–Mediatech INC, Manassas, VA) were added to each well and the plate was incubated at 50 °C for five min, 21 °C for 15 s, 50 °C for 1.5 min, and 21 °C for 15 s. The water was then removed from each well and discarded.

### Malaria genus PCR based on the 18s-rRNA (direct nested PCR)

**Fig. 1:**
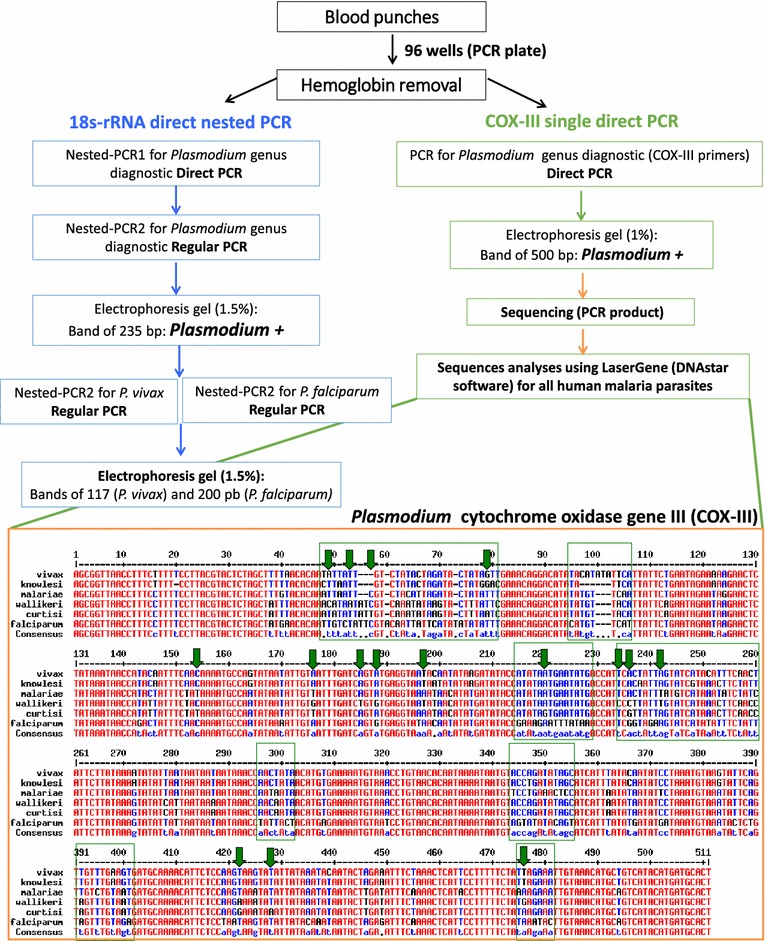
Flowchart of PCR techniques and alignment of the *Plasmodium* cytochrome oxidase gene III PCR products. For the cytochrome oxidase III (COX-III) gene the expected sizes of PCR fragments are: *P. vivax* (506 bp), *P. falciparum* (508 bp), *P. malariae* (504 bp), *P. knowlesi* (499 bp), *P. ovale wallikeri* and *P. ovale* *curtisi* (506 bp). With the COX-III gene sequence alignment, 140 polymorphisms (28 % of nucleotides) provide information for species diagnosis (there are eight blocks of highly polymorphic regions). For differentiation between *P. ovale wallikeri* and *P. ovale curtisi,* 16 SNPs were found (*green arrows*). When using 18s-rRNA nested PCR, additional Nest2 PCRs with species-specific primers are required for diagnosis of *P. malariae, P. ovale wallikeri, P. ovale curtisi* and *P. knowlesi*

A direct nested PCR strategy targeting the 18s-rRNA gene using the Blood Phusion Direct PCR Kit (Thermo Scientific, Waltham, MA) as described by Fuehrer et al. [17] was followed with modifications (Table 1). For the nest1 protocol, a total master-mix volume of 25 µL was added directly to the wells containing blood spots (Fig. 1, Table 1). After nest1 completion, the microplate was centrifuged at room temperature at 1000 *G* for 3 min. 3 µL of nest1 PCR product were transferred to a new microplate in order to perform the nest2 reaction (Fig. 1, Table 1) with a final volume of 20 µL. Nest2 PCR products were analysed by gel electrophoresis in a 1.5 % agarose gel stained with ethidium bromide (Sigma-Aldrich, St. Louis, MO).

**Table 1:** Summary of PCR conditions used in the 18s-rRNA and COX-III PCR strategies for human malaria diagnosis

Approach	Primers	Reagents final concentration	Cycling parameters
18s rRNA genus specific DIRECT PCR nest 1	rPLU1 TCAAAGATTAAGCCATGCAAGTGA rPLU5 CCTGTTGTTGCCTTAAACTTC	1X blood phusion buffer, 1 mM of primers, 1.0 mM of MgCl_2_ and 0.25 µL of phusion blood II DNA polymerase	98 °C for 4 min; 30 cycles of 94 °C for 1 min, 65° for 2 min and 72 °C for 2 min, and 72 °C for 4 min
18s rRNA genus specific PCR nest 2	rPLU3 TTTTTATAAGGATAACTACGGAAAAGCTGT rPLU4 TACCCGTCATAGCCATGTTAGGCCAATACC	1X PCR buffer, dNTPmix (200 uM each), 1 mM of primers, 1.0 mM of MgCl_2_ 50 mM and 0.15 µL of Taq DNA polymerase	94 °C for 4 min; 35 cycles of 94 °C for 30 s, 62° for 1 min and 72 °C for 1 min, and 72 °C for 4 min
18s rRNA species specific *P. vivax* nest 2	rVIV1 CGCTTCTAGCTTAATCCACATAACTGATAC rVIV2 ACTTCCAAGCCGAAGCAAAGAAAGTCCTTA	1X PCR buffer, dNTPmix (200 uM each), 1 mM of primers, 1.0 mM of MgCl_2_ 50 mM and 0.15 µL of Taq DNA polymerase	94 °C for 4 min; 35 cycles of 94 °C for 30 s, 58° for 1 min and 72 °C for 1 min, and 72 °C for 4 min
18s rRNA species specific *P. falciparum* nest 2	rFAL1 TTAAACTGGTTTGGGAAAACCAAATATATT rFAL2 ACACAATGAACTCAATCATGACTACCCGTC	1X PCR buffer, dNTPmix (200 uM each), 1 mM of primers, 1.0 mM of MgCl_2_ 50 mM and 0.15 µL of Taq DNA polymerase	94 °C for 4 min; 35 cycles of 94 °C for 30 s, 58° for 1 min and 72 °C for 1 min, and 72 °C for 4 min
COX-III DIRECT PCR nest1	Plas_COX3_F TGATAGCGGTTAACCTTTCYTTTTCCTTACG Plas_COX3_R CTGTTATCCCCGGCGAACCTTCTTAC	1X blood phusion buffer, 1 mM of primers, and 0.25 µL of phusion blood II DNA polymerase	98 °C for 4 min; 35 cycles of 98 °C for 1 s, 70° for ×5 s and 72 °C for 30 s, and 72 °C for 2 min
COX-III nest2	shortCOX-III_F *AGCGGTTAACCTTTC* *T* *TTTTCCTTACG* shortCOXIII_R AGTGCATCATGTATGACAGCATGTTTACA	1X PCR buffer, dNTPmix (200 uM each), 0.5 mM of primers, 1.5 uM of MgCl_2_ 50 mM and 0.2 µL of Taq DNA polymerase	94 °C for 5 min; 40 cycles of 94 °C for 1 min, 62° for 1 min and 72 °C for 90 s, and 72 °C for 10 min
COX-III Single DIRECT PCR	shortCOXIII_F *AGCGGTTAACCTTTC* *T* *TTTTCCTTACG* shortCOXIII_R AGTGCATCATGTATGACAGCATGTTTACA	1X blood phusion buffer, 1 mM of primers, and 0.30 µL of phusion blood II DNA polymerase	98 °C for 4 min; 70 cycles of 98 °C for 1 s, 70° for ×5 s and 72 °C for 35 s, and 72 °C for 10 min

## 18s-rRNA PCR for *Plasmodium vivax* and *Plasmodium falciparum* diagnosis

Malaria-species diagnosis [17] (nest2 PCR) was performed on positive samples from the malaria-genus PCR (presence of a 235 bp band), on the reference strains serial dilutions and on 21 positive control samples. As *P. vivax* (band of 121 bp) and *P. falciparum* (band of 200 bp) are the most prevalent species worldwide, separate rounds of nest2 PCRs were performed for diagnosis of both species using 3 µL of nest1 PCR product and a master-mix volume of 15 µL (Fig. 1, Table 1). Nest2 PCR products were analysed as above using a 2.0 % agarose gel. All 18s-rRNA PCR assays were amplified on an Eppendorf Master Cycler (Eppendorf) or GeneAmp PCR System 9700 (Applied Biosystems) machines.

### Cytochrome oxidase III primer design

The mitochondrial genome sequences (~5900 nucleotides) from *P. vivax* [GenBank: KF668441.1]*, P. falciparum* [GenBank M99416.1]*, Plasmodium malariae* [GenBank AB489194.1]*, Plasmodium ovale wallikeri* [GenBank HQ712053.1], *Plasmodium ovalecurtisi* [GenBank HQ712052.1], and *P. knowlesi* [GenBank AY722797.1] were aligned using MultAlin software [33]. A visual inspection of the alignment demonstrated several blocks of polymorphic sequences delimitated by conserved sequences among the *Plasmodium* species around the cytochrome oxidase III (COX-III) gene (Fig. 1). A set of primers, plas_cox3F/R (for nest1) and short_COX-III F/R (for nest2) (Table 1), were designed with the highest stringency in parameters using the DNAstar lasergene^®^ 11 software (DNAstar Inc. Madison WI). The annealing temperatures for these primers were determined using the manufacturer’s instructions [34].

### COX-III Nested and single direct PCR

Haemoglobin was removed as described. For the nest1 COX-III direct PCR the final reaction volume was 25 μL (Table 1). A nest2 PCR was then performed using 2 uL of the nest1 PCR product in a final reaction volume of 25 μL. For the single direct PCR reactions, the primers short COX-III F/R were used in a 25 μL final volume (Fig. 1, Table 1). PCR products were visualized on a 1 % gel stained with SYBR^®^safe (Invitrogen, Carlsbad, CA).

### Mixed infection test using the fast COX-III PCR

To estimate the performance of the single direct COX-III PCR on simulated mixed infections, the genomic DNA from *P. falciparum* (HB3 strain) and *P. vivax* (Miami strain) cultures were extracted following the protocol described in the E.Z.N.A. Blood DNA Mini Kit (Omega Bio-Tek, Norcross, GA). The concentration of the extracted DNA was determined using the Nanodrop 2000 (Thermo Scientific, Waltham, MA). Eight serial dilutions with a 1:6 factor were prepared for each species DNA. Serial dilutions from one species (from the highest to the lowest) were mixed with the serial dilutions from the other species (from the lowest to the highest) resembling different concentrations of DNAs found in *Plasmodium* spp. mixed infections (Table 2).

**Table 2:** Analyses of PCR products (COX-III single direct-PCR) of *Plasmodium* spp positive-controls and dilution performance of simulated mixed infections

*Plasmodium* spp.	BLAST result	
	% Identity	E value
*Plasmodium* spp. control (positive) samples		
*P. falciparum* (HB3 strain)	99.8	0.0
*P. vivax* (AMRU1 strain)	100	0.0
*P. malariae* (From the field)	99.8	0.0
*P. ovale wallikeri* (I.D 5463)	99.3	0.0
*P. ovale curtisi* (From the field)	98.4	3.2E−113
*P. knowlesi* (Malayan Strain)	100	8.1E−180
Serial dilutions of mixed samples in ng (higher concentration of *P. vivax* Miami strain)		
3.3 E−01 *P. vivax* + 1.1E−06 of *P. falciparum*	98.5 *P. vivax*	2.9E−93
5.5 E−02 *P. vivax* + 7.14E−06 of *P. falciparum*		
9.2 E−03 *P. vivax* + 4.2E−05 of *P. falciparum*		
1.5 E−04 *P. vivax* + 2.5 E−04 of *P. falciparum*		
Serial dilutions of mixed samples in ng (higher concentration of *P. falciparum* HB3 strain)		
2.5 E−04 *P. vivax* +1.5 E−04 of *P. falciparum*	99.5 *P. falciparum*	9.4E−100
4.2E−05 *P. vivax* + 9.2 E−03 of *P. falciparum*		
7.14E−06 *P. vivax* + 5.5 E−02 of *P. falciparum*		
1.1E−06 *P. vivax* + 3.3 E−01 of *P. falciparum*		

Serial dilutions 1:6 of eight different mixed concentration of DNA (higher for one species and lower for the other, and vice versa)

### Sequencing reactions for species diagnosis

A set of 100 predictive positive samples for the 18s-rRNA genus malaria diagnosis (with PCR product around 235 bp PCR product), and 20 from the species malaria *P. vivax* PCR (approx. 121 bp) were excised from gels and purified using the QIAquick gel extraction kit (QIAGEN, Valencia, CA) and sequenced with the rPLU3, Plasmo_int_2REV (3′ CATCAAAAGCTGATAGGTCAGA 5′–200 bp sequence length) and VIV1 primers respectively (Table 1). Eight microliter of PCR product from positive samples from COX-III single direct PCR reactions were purified with the ExoSAP-IT PCR clean-up kit (Affymetrix, Santa Clara, CA) and sequenced with the short_COX-IIIF primer. All sequencing reactions were performed on a MicroAmp Optical 96-well plate (Life Technologies) following the protocol described in the BigDye^®^ Terminator V3.1 kit (Applied Biosystems). The BigDye^®^ was precipitated and samples were re-suspended in 10 µL of Hi-Di Formamide and sequenced on an ABI 3730XL 96-capillary sequencer. Analyses of sequences were performed using the DNASTAR Lasergene ^®^ 11 software (DNAstar Inc. Madison WI).

## Results

### Lowest parasitaemia detected and validation assay on positive blood samples

**Fig. 2:**
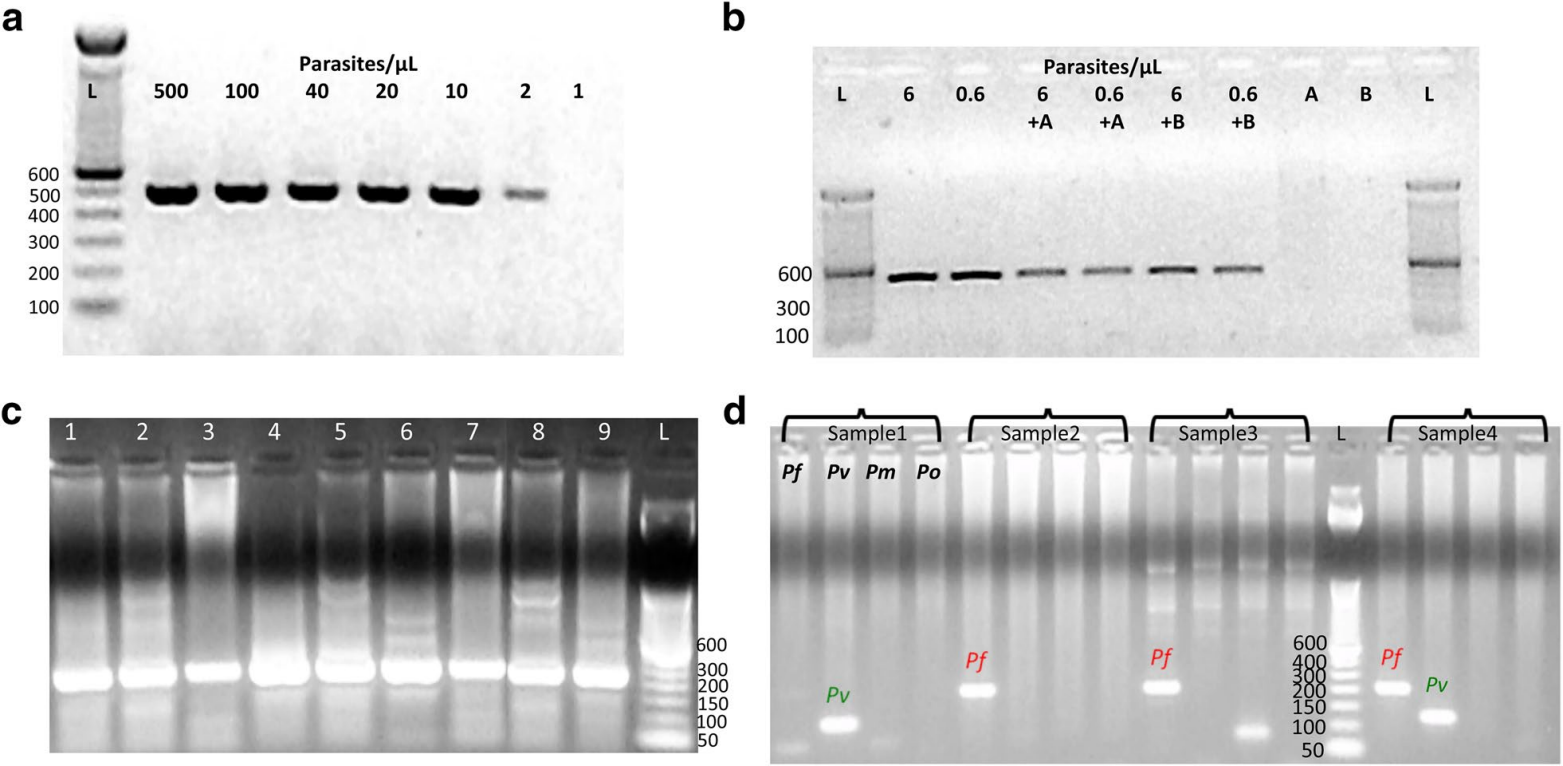
Lowest parasitaemia detected for the COX-III single direct-PCR and the 18s-rRNA nested-PCR on positive controls. **a** The lowest for *P. vivax* was 2 parasites/μL using the COX-III based PCR (expected fragment around 500 bp). **b** For *P. falciparum* (with COX-III PCR) was 0.6 parasites/μL. Adding one blood spot from healthy donors A and B to the parasite dilutions did not affect the PCR performance. Donor’s blood did not amplify any bands. **c** Performance of the 18s-rRNA genus-malaria nested PCR on nine positive control samples collected in the field (band of 235 bp). Non-specific bands are present. **d** *Plasmodium vivax* (121 bp), *P. falciparum* (200 bp) and a mixed infection detected in positive controls using the species-specific nested PCR

The lowest parasitaemia detected by PCR diagnostic assays were estimated using serial dilutions from parasite reference strains ranging from 500 to 0.06 parasites/μL. The lowest parasitaemia consistently detected for *P. falciparum* and *P. vivax* with the 18s-rRNA direct nested PCR (in 4/4 replicates) were 2 and 10 parasites/μL, respectively, while for the single direct COX-III PCR was 0.6 parasite/μL for *P. falciparum* and 2 parasites/μL for *P. vivax* (in 6/6 replicates) (Fig. 2a, b). Interestingly, the COX-III single direct PCR performed similar to or better than the nested COX-III PCR (data not shown). Since the COX-III single direct PCR was found to be simpler, shorter and more sensitive, the nested COX-III PCR was not analysed further. The addition of extra blood spots from negative malaria donors into a reaction did not affect the performance of the single direct COX-III PCR at the lowest *P. falciparum* detected parasitaemia (Fig. 2b).

When the 18s-rRNA nested PCR was validated with the 21 known positive blood samples, the test showed that all samples were positive with the genus malaria PCR (Fig. 2c) and with the species diagnosis as follows: *P. falciparum* (n = 7), *P. vivax* (n = 5), *P. malariae* (n = 1), *P. ovale* (n = 1) and mixed infection (n = 4) *(*Fig. 2d), and three samples did not amplify with any of the species-specific primers (Table 3). The COX-III single direct PCR amplified the expected band of 500 bp, and sequenced all the human *Plasmodium* parasites used as controls, including all the 21 known positive blood samples (100 % concordance with the 18s-rRNA genus malaria PCR) (Tables 2, 3). When compared with the 18s-rRNA nested PCR, the COX-III sequencing results confirmed species identification results for 14 samples and identified the missed three as *P. malariae;* the predicted four mixed infections were identified as mono-infections by either *P. vivax* or *P. falciparum* (Table 3).

**Table 3:** Comparison of malaria diagnosis on 21 positive samples based on the COX-III single direct-PCR versus the 18s-rRNA nested PCRs

Sample code	PCR fragment of COX-III	PCR fragment of 18s-rRNA		Agreement
		Genus	Species	
IN01	*P. malariae*	+	Negative^a^	No
IN02	*P. falciparum*	+	*P. falciparum*	Yes
IN03	*P. falciparum*	+	*P. falciparum*	Yes
IN04	*P. falciparum*	+	*P. falciparum*	Yes
IN05	*P. vivax*	+	*P. vivax*	Yes
IN06	*P. vivax*	+	*P. vivax*	Yes
IN07	*P. vivax*	+	Mixed infection^*b*^	No
IN08	*P. falciparum*	+	*P. falciparum*	Yes
IN09	*P. vivax*	+	*P. vivax*	Yes
IN10	*P. falciparum*	+	*P. falciparum*	Yes
IN11	*P. vivax*	+	Mixed infection^b, c^	No
IN12	*P. vivax*	+	Mixed infection^b^	No
IN13	*P. malariae*	+	*P. malariae*	Yes
IN14	*P. falciparum*	+	Mixed infection^*b*^	No
IN15	*P. ovale curtisi*	+	*P. ovale*	Yes
IN16	*P. malariae*	+	Negative^*a*^	No
IN17	*P. vivax*	+	*P. vivax*	Yes
IN18	*P. vivax*	+	*P. vivax*	Yes
IN19	*P. malariae*	+	Negative^*a*^	No
IN20	*P. falciparum*	+	*P. falciparum*	Yes
IN21	*P. falciparum*	+	*P. falciparum*	Yes

^a^ Negative results was obtain in two independent experiments

^b^ Mixed infections were due to *P. falciparum* and *P. vivax*

^c^ A second technical replicate of this sample was showed as a mono-infection with *P. vivax*

### Mixed infection test using the fast COX-III PCR

The performance of the COX-III single direct PCR on simulated mixed infections was tested using a range from 0.33 to 1.2E-06 ng of DNA from *P. falciparum* (HB3 strain) and *P. vivax* (Miami strain) (Table 2). For all the different ratios of DNA concentrations in the simulated mixed-infection, the band of about 500 bp was obtained in four independent assays. Sequencing of the COX-III PCR products identified the malaria parasite (*P. vivax* or *P. falciparum*) that had the higher concentration of DNA for a given simulated mixed-infection (Table 2).

### Validation of protocols on field samples

#### 18s-rRNA direct nested PCR

**Fig. 3:**
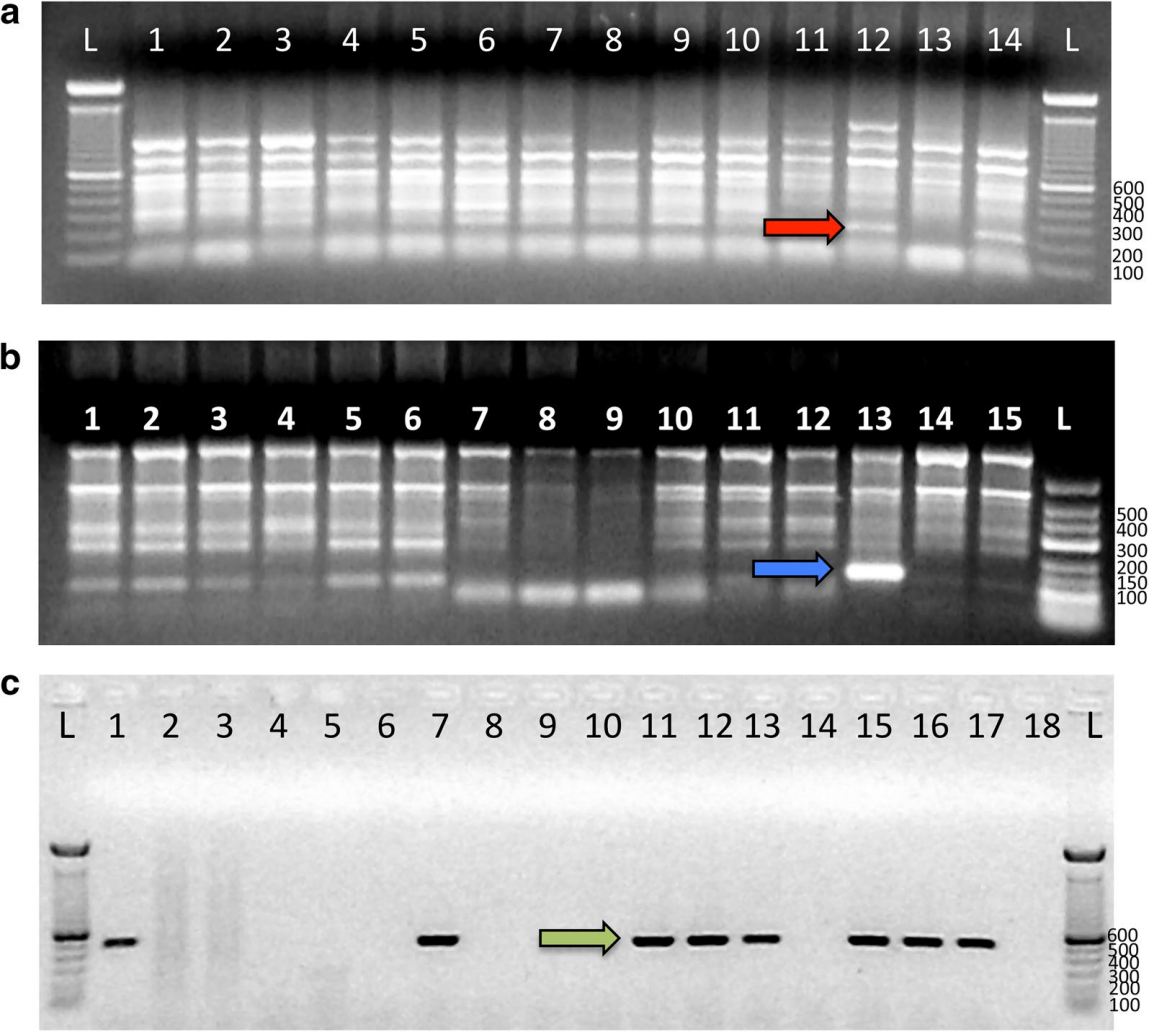
Performance of the 18s-rRNA nested-PCR and COX-III single direct-PCR on samples from the Solomon Islands. **a** Strong non-specific bands in 14 samples were amplified when the genus-malaria 18s-rRNA nested PCR was used. The expected diagnostic band for *Plasmodium* spp is 235 bp (*red arrow*). **b** Nonspecific bands close to the 121 bp diagnostic bands for *P. vivax* (*blue arrow*); only the sample 13 shown a robust band. **c** Eighteen samples tested for *Plasmodium* spp using the COX-III gene, a band of approximately 500 bp (*green arrow*) represent positive samples

A total of 3833 samples were screened for malaria using the modified 18s-rRNA direct nested PCR (Table 1, Fig. 1). There were 636 (16.6 %) *Plasmodium* positive samples with the 235 bp diagnostic band. Faint bands around 235 bp were found singularly or accompanied with strong bands usually above 300 bp without evidence of contamination in the negative control wells. These non-specific amplifications were seen in almost 85 % of all *Plasmodium* genus PCRs performed (Fig. 3a).

There was a lack of concordance between the genus and species PCRs using the 18s-rRNA direct nested diagnostic assay. All *Plasmodium* PCR positive samples were only tested for *P. falciparum* and *P. vivax* (Fig. 1). About 30 % of the positive genus-*Plasmodium* samples were positive by species-specific PCR. A total of 179 infections (95.2 %) were diagnosed as *P. vivax* and nine (4.8 %) by *P. falciparum* (overall, 4.9 % of samples were positive). Again, non-specific bands were seen above 300 bp and close to the diagnostic band for *P. vivax* (Fig. 3b) and *P. falciparum.* The 448/636 samples that did not amplify any of the malaria species, were rerun with the *P. vivax* specific primers (as this is the most prevalent species in the tested samples). For this set of samples amplifications were not obtained.

A total of 100 and 20 predicted positive samples were sequenced to verify *Plasmodium* spp. and *P. vivax* diagnosis respectively (Fig. 3b). For the sequencing reactions with Plasmo_int_2REV or rPLU4 primers, only seven samples were identified as *P. vivax.* For the remaining 93 samples, the sequencing results were inconclusive or did not have similarity with any sequence from GenBank. For the 20 sequencing reactions run with VIV1 primer, eight samples showed similarity with *P. vivax*, four were human DNA, and eight had no similarity with any sequence.

### COX-III single direct PCR

The COX-III single direct PCR approach was used for *Plasmodium* spp. detection in the 3833 samples. The diagnostic band of 500 bp was seen in 79/3833 samples (2 % of positive samples) (Fig. 3c). In these samples, a total of 39 and 40 samples were positive from the previously 640 predicted positive and negative samples using the 18s-rRNA direct nested PCR, respectively. When comparing the 3833 set of samples with both PCR approaches (18s-rRNA and COX-III), the COX-III direct PCR detected only 6 % of the 640 predicted positives by the nested PCR.

Overall, from the 79 sequenced samples with the short_COX-IIIF primer, 76 % were *P. vivax*, 12 % were *P. falciparum, 8* *%* were *P. ovale wallikeri* and 4 % were *P. malariae*. A further 3506 blood spots were also tested with this protocol with similar results (data not shown). This method also generated clearer diagnostic bands without non-specific bands (Fig. 3c).

## Discussion

Since 1993, the 18s-rRNA gene has been extensively used in different PCR assays for malaria diagnosis [14, 15, 30, 35–38]. The nested PCR approach has been the most widely used and often regarded as the standard PCR diagnostic method (primarily using high purity DNA and the original protocol), when comparing new PCR-based diagnostic approaches [17, 19, 23, 39, 40]. The 18s-rRNA nested PCR was not designed to be used with crude blood spots in a direct PCR strategy.

In the present study, the limit of detection or sensitivity, using the 18s-rRNA direct nested PCR was 2 and 10 parasites/μL for *P. falciparum* and *P. vivax* respectively, which is comparable with previous studies [16–18]. Only 18 of 21 positive control blood spot samples were positives for genus and species, with the three *P. malariae* samples being not detected by the18s-rRNA nested PCR. Whereas, all 21 positive control samples were detected by the COX-III single direct PCR.

Several studies have described inconsistencies in malaria positivity rates when species-specific reactions are performed using the 18s-rRNA gene [14, 19, 41–43] (Table 4). Several problems were identified when using the nested PCR to analyse the field samples: (a) 75 % of positive samples gave discordant diagnoses when genus and species-specific results were compared; (b) it was not possible to confirm predictive positive samples (genus and species PCR) with sequencing; and (c) 40 samples positive with the COX-III single direct PCR were not identified with the18s-rRNA genus nested PCR. Possible explanations for the results from the nested protocol include the following:

**Table 4:** Summary of inconclusive results when the 18s-rRNA species-specific primers are used for malaria diagnosis

Location	Proportion of inconsistent species-specific identification over total predicted positives for genus (%)	Source material (DNA extraction method)	References
Cambodia	12/256 (4.7)^a^	Blood spots (commercial kit and instagene)	[19]
Tanzania	21/76 (27.6)^b^	Whole blood (commercial kit)	[41]
Brazil	13/34 (38)^c^	Whole blood (commercial kit)	[43]
Malaysia	3/46 (6.5)^a^	Blood spots (chelex)	[14]
Bangladesh	2/97 (2)^a^	Blood spots (direct PCR) and (instagene-chelex)	[17]
Nigeria	37/285 (13)^d^	Blood spots (commercial kit)	[21]

^**a**^ 18s-rRNA genus were positive but no species identified by nested PCR

^**b**^ The genus diagnostic was based on mitochondrial DNA while specie-specific diagnosis targeted the 18s rRNA

^**c**^ 13 samples were tested by quadruplicate with species-specific primers without reproducible results

^**d**^ Results from *P. falciparum* specific primers (rFAL1/rFAL2) were compared against composite reference results

1. Amplification of non-specific bands: The amplification of non-specific bands in the nested PCR diagnostic have been described [23, 44] and reported with the primers PLF, UNR and VIR (also targeting the 18s-rRNA gene) [45]. Sequencing of a subset of predicted positive samples for genus and species did not produce readable data (93 % and 55 % of the samples tested respectively), suggesting non-specific amplification. Without evidence of contamination in the negative controls, the presence of non-specific bands near or at the expected diagnostic band size increased the number of false positives.
2. Lower DNA yield from blood spots: The number of parasites can differ by more than 15 % between different punches from the same bloodspot [22]. It is also suggested that samples analysed from dried blood spots report half the *Plasmodium* positive rate compared with analyses using whole blood samples [23]. Samples in this study are from a low transmission setting (Western Province, Solomon Islands) under malaria pre-elimination phase, and may have extremely low parasite densities, which may fail to amplify (Monte Carlo effect) [43] or produce inconsistent or unspecific amplifications with a protocol not sensitive enough.
3. The methodology described by Fuehrer et al. [17] utilized phusion blood DNA polymerase, while in this study the phusion blood II DNA polymerase was used. The phusion blood II DNA polymerase in the direct PCR kit contains an improved affibody molecule which allows for lower annealing temperatures and wider range of PCR primers, this may explain the non-specific bands seen here, and may require extra optimization of temperatures with a gradient cycler. This issue was not seen when the phusion blood DNA polymerase was used.
4. Presence of blood inhibitors: Blood has potent PCR inhibitors such as haemoglobin, hemin, immunoglobulin G and lactoferrin [46, 47]. In PCR reactions, the Taq polymerase enzyme is completely inhibited in the presence of 0.004 % (v/v) of human blood [48]. Blood compounds transferred from the first PCR product (nest1) to the second (nest2) PCR reaction might affect amplification of target DNA.

The lowest parasitaemia detected with the COX-III single direct PCR (≤2 parasites/ul found in *P. vivax* and *P. falciparum*) is comparable with malaria diagnostic techniques based on mitochondrial or *Pfr364* and *Pvr47* genes, LAMP isothermal reactions, real time PCR and single PCRs [23, 39, 49, 50]; and better when compared with 18s-rRNA isothermal reactions, real time and multiplex PCRs [37, 51, 52]. Differences in sensitivity are correlated with: (1) higher copy numbers of COX-III and other mitochondrial genes (up to 150) in comparison with up to 8 copies in the 18s-rRNA gene [40], (2) stringent design of primers (100 and 66 % similarity with *Plasmodium* and human DNA respectively) and (3) the robustness and accuracy of the phusion blood II DNA polymerase used in the direct PCR. In a recent manuscript, a new efficient PCR (one round) based on the COX-III gene (using purified DNA) for *Plasmodium* genus detection was described, with nested PCRs required for *Plasmodium* species identification [40].

The use of direct PCR avoids DNA extraction/purification steps, thereby retaining all DNA in the sample while reducing the risk of cross-contamination. The practicality and shorter time-to-result with the single COX-III direct PCR (1 h) compared with other PCR assays make this technique suitable for large-scale application and high-throughput analyses, while also dramatically reducing time, effort and resources. The single COX-III direct PCR is also less expensive. The cost of malaria diagnosis of 96 blood spots with 10 positive samples (for species identification) using the single direct COX-III PCR (including only the single COX-III PCRs, PCR product purification and sequencing) can be up to a quarter of the cost when compared with the 18s-rRNA nested PCR (192 PCRs for genus diagnosis and 50 for species diagnosis) as described here, the COX-III PCR assay developed by Isozumi et al. [40], and a 18s-rRNA real time PCR strategy [39].

Only a few protocols have been designed to discriminate between the species *P. ovale curtisi* (classic type) and *P. ovale wallikeri* (variant type) [18, 53]. There are 97 predicted polymorphisms (88 SNPs and 9 INDELs) between the *P. ovale wallikeri* (HQ712053.1) and *P. ovale curtisi* (HQ712052.1) mitochondrial genomes (5850 bp). The COX-III single direct PCR amplifies a 506 bp fragment, which has 16 SNPs (16.5 % of the total polymorphisms) accounting for an accurate diagnosis for both species. A positive control sample from the field was identified as *P. ovale curtisi* while *P. ovale wallikeri* was found in a reference control and three samples from the larger dataset (Tables 2, 3).

Mixed *Plasmodium* infection diagnosis is rare (up to 4 %) with microscopy but common (up to 65 %) when PCR techniques are used [54]. The single COX-III direct PCR was able to detect only one species in the three predicted mixed infections detected by the nested 18s-rRNA in positive control samples (Table 3). Also, the new technique consistently amplified parasite DNA in mock mixed infections (*P. vivax* and *P. falciparum*), but the sequencing reactions only detected the predominant infection (Table 2). This is consistent with previous studies when the difference of DNA concentration is >10-fold between parasites in a mixed infection, only the most abundant parasite is amplified when multiplex PCR or real time techniques are used [51]. The use of specific primers for each *Plasmodium* species may be the most precise approach for determining species in mixed malaria infections.

## Conclusions

In conclusion, a cheaper, faster, and more sensitive PCR/sequencing method to accurately identify all human malaria parasites (including the *P. ovale* sub-species) was developed and tested on more than 7000 blood spots, using the cytochrome oxidase III (COX-III) as target. This method will simplify screening samples as well as minimize chances of contamination as DNA extraction and nested PCR reactions are not required. This sensitive diagnostic tool will be useful in detecting submicroscopic and/or asymptomatic malaria when active screening a large number of subjects.

## Abbreviations

18s-rRNA: 18 small subunit ribosomal RNA
ACT: artemisinine combinatory therapy
COX-III: cytochrome oxidase III
IRS: indoor residual spraying
ITNs: insecticide-treated bednets
mm: millimeter
MR4: malaria Research and Reference Reagent Resource Center
MSA-2: merozoite surface antigen 2
ng: nanograms
RDT: rapid diagnostic test
Sec: seconds
STEVOR: subtelomeric variable open reading frame
µL: microlitre
